# Bacterial Diversity and Antibiotic Susceptibility of *Sparus aurata* from Aquaculture

**DOI:** 10.3390/microorganisms8091343

**Published:** 2020-09-02

**Authors:** Vanessa Salgueiro, Vera Manageiro, Narcisa M. Bandarra, Lígia Reis, Eugénia Ferreira, Manuela Caniça

**Affiliations:** 1 National Reference Laboratory of Antibiotic Resistances and Healthcare Associated Infections (NRL-AMR-HAI), Department of Infectious Diseases, National Institute of Health Dr. Ricardo Jorge, 1649-016 Lisbon, Portugal; vanessa.salgueiro@insa.min-saude.pt (V.S.); vera.manageiro@insa.min-saude.pt (V.M.); ligia.reis@insa.min-saude.pt (L.R.); eugenia.ferreira@insa.min-saude.pt (E.F.); 2 Centre for the Studies of Animal Science, Institute of Agrarian and Agri-Food Sciences and Technologies, Oporto University, 4051-401 Oporto, Portugal; 3 Department of Sea and Marine Resources, Portuguese Institute for the Sea and Atmosphere (IPMA, IP), 1749-077 Lisbon, Portugal; narcisa@ipma.pt

**Keywords:** *Sparus aurata*, aquaculture, antibiotic resistance, *qnrB19*, One Health

## Abstract

In a world where the population continues to increase and the volume of fishing catches stagnates or even falls, the aquaculture sector has great growth potential. This study aimed to contribute to the depth of knowledge of the diversity of bacterial species found in *Sparus aurata* collected from a fish farm and to understand which profiles of diminished susceptibility to antibiotics would be found in these bacteria that might be disseminated in the environment. One hundred thirty-six bacterial strains were recovered from the *S. aurata* samples. These strains belonged to *Bacillaceae*, *Bacillales* Family XII. *Incertae Sedis*, *Comamonadaceae*, *Enterobacteriaceae*, *Enterococcaceae*, *Erwiniaceae*, *Micrococcaceae*, *Pseudomonadaceae* and *Staphylococcaceae* families. *Enterobacter* sp. was more frequently found in gills, intestine and skin groups than in muscle groups (*p* ≤ 0.01). Antibiotic susceptibility tests found that non-susceptibility to phenicols was significantly higher in gills, intestine and skin samples (45%) than in muscle samples (24%) (*p* ≤ 0.01) and was the most frequently found non-susceptibility in both groups of samples. The group of *Enterobacteriaceae* from muscles presented less decreased susceptibility to florfenicol (44%) than in the group of gills, intestine and skin samples (76%). We found decreased susceptibilities to β-lactams and glycopeptides in the *Bacillaceae* family, to quinolones and mupirocin in the *Staphylococcaceae* family, and mostly to β-lactams, phenicols and quinolones in the *Enterobacteriaceae* and *Pseudomonadaceae* families. Seven *Enterobacter* spp. and five *Pseudomonas* spp. strains showed non-susceptibility to ertapenem and meropenem, respectively, which is of concern because they are antibiotics used as a last resort in serious clinical infections. To our knowledge, this is the first description of species *Exiguobacterium acetylicum*, *Klebsiella michiganensis*, *Lelliottia* sp. and *Pantoea vagans* associated with *S. aurata* (excluding cases where these bacteria are used as probiotics) and of plasmid-mediated quinolone resistance *qnrB19*-producing *Leclercia adecarboxylata* strain. The non-synonymous G385T and C402A mutations at *parC* gene (within quinolone resistance-determining regions) were also identified in a *Klebsiella pneumoniae*, revealing decreased susceptibility to ciprofloxacin. In this study, we found not only bacteria from the natural microbiota of fish but also pathogenic bacteria associated with fish and humans. Several antibiotics for which decreased susceptibility was found here are integrated into the World Health Organization list of “critically important antimicrobials” and “highly important antimicrobials” for human medicine.

## 1. Introduction

In the last 30 years, global production of the aquaculture sector has increased and today represents nearly half of the fish consumed worldwide, with China as the main producer. This was a consequence of higher demand due to the reduction or stagnation of fishing catches and an increasing world population [1].

Portugal is the third major consumer of fish, in Europe [2]. Although aquaculture represents a small portion of this consumption, this sector has grown in the last decades and is expected to increase in the coming years. This is a country with favorable conditions for aquaculture, where the main production is of bivalve mollusks, and extensive and intensive systems are predominant. Tanks for fish production represent about 5% of all Portuguese aquaculture infrastructures (in 2012), and turbot (*Scophthalmus maximus*), European seabass (*Dicentrarchus labrax*) and gilthead seabream (*Sparus aurata*) are main species produced [3]. *S. aurata* is a species belonging to the *Sparidae* family and *Perciformes* order [4] and is often found in shallow waters and is sensitive to water temperature. The gilthead seabream has great commercial importance in Europe, representing one of the main species cultivated in this continent [5].

However, despite all the advantages, aquaculture production can have a negative impact, specifically in degradation of natural resources and rising of antibiotic use [1,6]. A higher density of fish in a specific area is usually related to an increase in stress conditions, leading to a predisposition for infectious diseases and a higher antibiotic consumption. Therefore, these environments can function as a reservoir of antibiotic resistance and/or antibiotic-resistant genes. In Europe, antibiotics for growth promotion are not authorized, and only eight antibiotics are allowed for prophylaxis and therapeutics: ampicillin, oxytetracycline, florfenicol, flumequine, oxolinic acid, sarafloxacin, erythromycin and sulphonamides associated with trimethoprim or ormetoprim. These antibiotics are mostly administered by oral-medicated feed or bath, which are the easiest methods to apply but expose both sick and healthy individuals to antibiotics and allow the accumulation of these substances in sediments and water. This contributes to selective pressure in bacteria from these environments [7,8].

In this contexts, this study aimed to contribute to a deeper knowledge of the diversity of bacterial species found in *S. aurata* from aquaculture and the respective antibiotic susceptibilities that can be disseminated in their environment. The correlation of antibiotic resistance with the World Health Organization (WHO) list of “critically important antimicrobials” and “highly important antimicrobials” for human medicine will also be established.

## 2. Materials and Methods

### 2.1. Sample Collection and Preparation

Five commercial-size *S. aurata* (500–1500 g) were collected in March 2018 in a land tank from a fish farming pilot station in the south of Portugal by the Portuguese Institute of Sea and Atmosphere. This station is located in the Ria Formosa Natural Park and is an integrated multi-trophic aquaculture with a semi-intensive system. The weight, furcal length, total length, and condition index of the 5-gilthead seabream were measured (Table 1). Each fish was divided into 4 samples (gills, intestine, muscle, and skin), that were frozen and transported on ice to the National Institute of Health Dr. Ricardo Jorge, where they were analyzed. This study includes the results of the testing for 5 samples of muscle (from fish 1, 2, 3, 4 and 5) and the gills, intestine and skin samples from fish 1 that were treated separately (excepting for some results presentation) (Figure S1).

### 2.2. Bacterial Isolation and Identification

Ten grams of each sample was homogenized in peptone water (Stomacher 80 Biomaster^®^, Seward, UK), incubated for 12 to 18 h at 37 °C and further diluted [10,11]. Each dilution was plated in selective media (MacConkey agar, Mannitol salt agar and UriSelect™4 chromogenic agar) and incubated for 18 to 20 h at 37 °C. Colonies with different morphology (to avoid duplications) were selected and DNA extracted, according to manufacturer’s instructions (MagNA Pure 96 Instrument, Roche, Manheim, Germany). Strains were identified by VITEK2 and amplification of the 16S rRNA gene, as already described [12].

### 2.3. Antibiotic Susceptibility Testing

Antibiotic susceptibility testing was performed by disk diffusion (Bio-Rad, Marnes-la-Coquette, France) and minimum inhibitory concentration (MIC) by in-house broth microdilution and E-test® (bioMérieux, Marcy l’Etoile, France). Different bacterial families were tested for different antibiotics (Table 2). 

For *Enterobacteriaceae* and *Erwiniaceae*, the antibiotics tested were amoxicillin/clavulanic acid, aztreonam, cefepime, cefotaxime, cefoxitin, ceftazidime, ertapenem, imipenem, meropenem, piperacillin/tazobactam, ciprofloxacin, trimethoprim/sulfamethoxazole, gentamicin, chloramphenicol, florfenicol, flumequine and oxytetracycline. For *Pseudomonadaceae*, the antibiotics tested were aztreonam, cefepime, ceftazidime, doripenem, ertapenem, imipenem, meropenem, piperacillin/tazobactam, ciprofloxacin, levofloxacin, amikacin, gentamicin, netilmicin, tobramycin, chloramphenicol, florfenicol, flumequine and oxytetracycline. On the other hand, for *Staphylococcaceae*, the antibiotics tested were cefoxitin, ciprofloxacin, levofloxacin, moxifloxacin, rifampicin, mupirocin, fusidic acid, daptomycin, linezolid, teicoplanin and vancomycin. For *Enterococcaceae*, the antibiotics tested were ampicillin, high concentration (HC) gentamicin, HC streptomycin, linezolid, teicoplanin and vancomycin. For *Bacillaceae*, the antibiotic tested was vancomycin.

For Gram-negative bacteria, the antibiogram was completed with (1) disc combination test (DCT or combined disk test, CDT) [13], which is based on the comparison between zone diameters of one disc of antibiotic alone and another with an inhibitor, here using cefotaxime (30 µg) and cefotaxime/clavulanic acid (30 µg + 10 µg) to search for the presence of extended-spectrum β-lactamase (ESBL-positive if there is a difference of ≥ 5 mm); (2) DCT to compare zone diameters of meropenem and meropenem/dipicolinic acid (1000 µg), to search for the presence of Metallo-β-lactamase (MBL-positive if ≥ 5 mm) [14]; (3) DCT with amoxicillin and amoxicillin/clavulanic acid plus cloxacillin (500 µg), to search for the presence of AmpC (positive for cephalosporinases if ≥ 5 mm) [13]; (4) double disc synergy test (DDST) with two discs containing predefined amounts of the β-lactam and the inhibitor, placed close to each other, here using boronic acid (300 µg) and carbapenems to search for any class A carbapenemase (positive if a synergy is observed between carbapenemes discs) [14]; (5) DDST to search for MBL when a synergy is observed between dipicolinic acid and carbapenemes [14]; (6) DDST to detect AmpC when a synergy is observed between boronic acid and third-generation cephalosporins and/or cloxacillin and cefoxitin/ceftazidime [13]; (7) temocillin disc to indicate the presence of an OXA-48 carbapenemase; and (8) faropenem disc to indicate the presence of carbapenemases, confirming the results from (4), (5) and (7) tests.

MIC_50_ and MIC_90_ were calculated for *Enterobacteriaceae* since it was the most represented family, as reported elsewhere [15]. MIC_50_ represents the MIC value that inhibits 50% of the strains tested (and is equivalent to the median MIC value), whereas MIC_90_ represents the MIC value that inhibits 90% of the strains tested. *Escherichia coli* strain ATCC 25922 was used as quality control for Gram-negative bacteria, whereas *Staphylococcus aureus* strain ATCC 25923 and *Enterococcus faecalis* ATCC 29212 were used for Gram-positive bacteria.

### 2.4. Statistical Analyses

Statistical analyses of the results were performed to detect positive or negative associations between fish samples (muscle vs. gills, intestine and skin) and each bacterial family/species and non-susceptibility to different antibiotic’s class (only factors identified as statistically significant are shown). Fisher exact test was used to assess differences in bacterial families/species/non-susceptibility to different antibiotic’s class between fish samples, and one-tailed *p*-values of ≤ 0.05 were considered to be statistically significant. Associations were established by calculation of odds ratios with 95% confidence intervals. The null hypothesis was rejected for *p*-values of ≤ 0.05. All statistical analyses were calculated using OpenEpi software, v. 3.01 [16].

### 2.5. Detection of Antibiotic Resistance Genes

The interpretation of the antibiotic susceptibility testing results guided the research of resistance genes by PCR (Polymerase Chain Reaction), and all the positive results were sequenced, as described elsewhere [12].

The genes *bla*_CTX-M_, *bla*_TEM_, *bla*_SHV_ and *bla*_OXA-1-type_ were investigated for Gram-negative strains that showed decreased susceptibility to β-lactams and/or demonstrated a positive result for the DCT and DDST [17,18]. The presence of genes *bla*_OXA-48_, *bla*_VIM_, *bla*_IMP-1-type_, *bla*_NDM_, *bla*_KPC_, *bla*_GES_ and *bla*_SME_ was studied for Gram-negative bacteria with decreased susceptibility to carbapenems and/or that demonstrated a positive result for the DCT and DDST, using primers described in this study for the first time (Table 3), and others previously described [19,20,21,22,23]. All Gram-negative strains with decreased susceptibility to quinolones were tested for *qnrA*, *qnrB*, *qnrC*, *qnrD*, *qnrS*, *aac(6’)-Ib* and *qepA* genes (Table 3) [24,25,26,27,28]. For one *Klebsiella pneumoniae* strain with decreased susceptibility to ciprofloxacin, which tested negative for the genes described previously, we searched for mutations in the *gyrA*, *gyrB*, *parC* and *parE* genes (Table 3) [29,30,31]. All Gram-negative bacteria with decreased susceptibility to quinolones but negative results for *qnr*, *aac(6’)-Ib* and *qepA* genes were investigated for the presence of *oqxAB* genes, with primers and PCR reactions conditions described elsewhere [12]. All Gram-negative strains were investigated for the presence of *mcr-1*, *mcr-2*, *mcr-3*, *mcr-4* and *mcr-5* genes as previously described [32].

Apart from the antibiotic susceptibility testing results, all *Staphylococcus* spp. were tested for the presence of *mecA*, *mecC*, *vanA*, *vanB* and *vanD* genes (Table 3) [33]. On the other hand, all *Enterococcus* spp. and three *Bacillus* spp. resistant to vancomycin were investigated for the presence of *vanA*, *vanB* and *vanD* genes. The cycling conditions for the PCR multiplex for detection of *vanA*, *vanB* and *vanD* genes were as follows: 1 cycle of denaturation at 94 °C for 10 min, followed by 30 cycles of denaturation at 94 °C for 30 s, annealing at 55 °C for 1 min and elongation at 72 °C for 1 min, and a final cycle of elongation at 72 °C for 10 min.

## 3. Results

### 3.1. Bacterial Diversity in S. aurata Samples

One hundred thirty-six bacterial strains were recovered from the total of *S. aurata* samples. Eighty-eight were Gram-negative bacteria, and 48 were Gram-positive bacteria. The results of VITEK2 and amplification of the 16S rRNA gene revealed that the majority of strains belonged to the *Enterobacteriaceae* family (55% in muscle samples and 60% in gills, intestine and skin samples), followed by *Staphylococcaceae* in muscle samples (16%) and *Bacillaceae* in gills, intestine and skin samples (17%) (Table 4). *Bacillales* Family XII. *Incertae Sedis*, *Comamonadaceae* and *Micrococcaceae* families were only found in muscle samples, whereas *Erwiniaceae* family was only found in gills samples. Nevertheless, these results do not represent statistically significant differences.

Within the most represented family, *Enterobacteriaceae*, we observed the following species of bacteria: *Enterobacter cloacae*, *Enterobacter hormaechei*, *Enterobacter* sp., *Klebsiella michiganensis*, *K. pneumoniae*, *Leclercia adecarboxylata*, *Lelliottia* sp. (in both groups of samples), *Citrobacter freundii*, *Citrobacter freundii* complex (only in muscle samples) and *E. coli* (only in gills sample). *Staphylococcaceae* family included *S. aureus*, *Staphylococcus haemolyticus*, *Staphylococcus pasteuri* (in both groups of samples), *Staphylococcus capitis*, *Staphylococcus epidermidis*, *Staphylococcus petrasii*, *Staphylococcus saprophyticus* and *Staphylococcus* sp. (only in muscle samples). *Bacillaceae* included *Bacillus cereus*, *Bacillus* sp. (in both groups of samples), *Bacillus pumilus*, *Bacillus thuringiensis* (only in muscle samples), *Bacillus amyloliquefaciens* and *Bacillus subtilis* species (only in gills, intestine and skin group of samples). *Pseudomonadaceae* included *Pseudomonas stutzeri* (in both groups of samples) and *Pseudomonas putida* (only in muscle samples). *Enterococcaceae* included *Enterococcus hirae* (in both groups of samples) and *Enterococcus faecalis* (only in gills sample). *Bacillales* Family XII. *Incertae Sedis* included *Exiguobacterium acetylicum* species. *Comamonadaceae* included *Comamonas aquatica* species. *Micrococcaceae* included *Kocuria rhizophila* species. Finally, *Erwiniaceae* family included *Pantoea vagans* species. Despite appearing in both groups of samples, *Enterobacter* sp. are more frequently found in gills, intestine and skin group than in muscle group (*p* ≤ 0.01; Table 5, Table S1). Statistically significant associations were not found for the other species analysed.

### 3.2. Phenotypic Characterization of the Bacterial Strains

In this study, non-susceptibility to phenicols was the most frequently found in both groups of samples, being significantly higher in gills, intestine and skin samples (45%; Table 6) than in muscle samples (24%) (*p* ≤ 0.01; Table 5). Non-susceptibility to β-lactams is the second most prevalent in both groups of samples (13% in muscles and 10% in gills, intestine and skin samples; Table 6). Decreased susceptibility to quinolones was also found in this study (7% in muscle and 5% in gills, intestine and skin samples). On the other hand, decreased susceptibility to glycopeptides and to mupirocin was only found in muscle samples.

Among *Enterobacteriaceae* strains, no differences were observed in MIC_50_ and MIC_90_ between the two groups of samples for the four antibiotics tested (Table 7). For each antibiotic, slight differences were registered between MIC_50_ and MIC_90_ (just 1-fold dilution). When comparing the decreased susceptibilities between the two groups of samples (muscle vs. gills, intestine and skin), major difference were found only in florfenicol, with the group of *Enterobacteriaceae* from muscles presenting 44% of nonsusceptible strains, while in the group of gills, intestine and skin samples, the values were higher, with 76% (Table 7).

In Table 8 is registered the decreased susceptibility profile for the 61 strains that had a non-susceptibility result for at least one antibiotic (including intrinsic non-susceptibilities). In Gram-positive bacteria, we found decreased susceptibilities to β-lactams and glycopeptides in the *Bacillaceae* family and to quinolones and mupirocin in the *Staphylococcaceae* family. In Gram-negative bacteria, *Enterobacteriaceae* family showed several decreased susceptibility profiles, with non-susceptibilities to β-lactams, phenicols and quinolones; the same non-susceptibilities to these antibiotic classes were found in strains from the *Pseudomonadaceae* family. The non-susceptibility to ertapenem of seven *Enterobacter* spp. strains and to the antipseudomonal carbapenem (meropenem) of five *Pseudomonas* spp. was found.

### 3.3. Genotypic Characterization

The search for resistance genes by PCR revealed a *qnrB19* gene in a *L. adecarboxylata* strain, with decreased susceptibility to ciprofloxacin (zone diameter = 24 mm). The analyses of the mutations present in *gyrA*, *gyrB*, *parC* and *parE* genes of a *K. pneumoniae* strain with decreased susceptibility to ciprofloxacin (zone diameter = 25 mm) showed the presence of non-synonymous mutations G385T (Ala129Ser in protein) and C402A (Ser134Arg in protein) in *parC* gene. No other resistance genes were identified in the studied bacteria among all the searched genes.

## 4. Discussion

The aquaculture sector has experienced a strong growth in recent decades and with it the number of studies to answer some concerns about the quality and safety of its products. Some studies have focused on the search of antibiotic residues in waters and/or sediments from fish farms, while others have focused on the search for specific pathogens, such as *E. coli*, not only in water and/or sediments but also in fish and shellfish [34,35,36,37,38,39]. This study aims to provide data, not only on the bacterial diversity in *S. aurata* from aquaculture, but also on antibiotic resistant genes that circulate in these environments. This information is crucial in the construction of science-based policies for the suitable use of antibiotics in aquaculture.

In this study, we found a very diverse bacterial population with 31 species that belonged to nine different families. Microbiota present in fish depends not only on the fish genetics and diet but is also determined by microbiota present in their environment, such as water and sediments. Microbiome composition is normally different between individual fish belonging to the same species but also varies between healthy and sick individuals, wherein the healthy individuals seem to have a higher diversity of bacterial population [40]. The condition index of fish belonging to this study varied between 1.56 and 2.04 (Table 1), meaning that these *S. aurata* were healthy (values higher than 1), possibly explaining the diversity found.

Of the species found in this study, only *Pseudomonas* spp. and *K. rhizophila* appeared to frequently cause diseases in fish, *K. rhizophila* being an emerging pathogen [41,42]. *Pseudomonas* spp. are ubiquitous in nature, and *P. fluorescens* is the most important species in fish infections (not found in this study), although *P. putida* had already been found in internal organs of fish and *P. stutzeri* in sediments of marine waters [43]. This genus is responsible for strawberry disease and septicaemia in some fish species [42]. *P. putida* and *P. stutzeri* are also opportunistic pathogens in humans and were already associated with bacteraemia, endocarditis, keratitis, meningitis, pneumonia, skin and soft tissue infections and urinary tract infections [44,45,46,47,48]. *Kocuria* spp. are Gram-positive and coccoid bacteria isolated from numerous environments: skin of mammals, marine sediments, soil and food [49,50,51,52,53]. Specifically, *K. rhizophila* causes a variety of lesions in fish and is responsible for a 50% mortality rate: exophthalmia, skin petechiae, increased skin melanization, liver congestion, inflammation of the intestine and hemorrhages [42]. Reports revealed that this species was already responsible for some infections in humans, mainly catheter-related bacteremia [54].

Other bacterial species from this study are known to be opportunistic pathogens in fish, some already described in *S. aurata*: *C. freundii* complex, *Staphylococcus* spp., *K. pneumoniae*, *E. faecalis* and *E. cloacae* [55,56,57,58,59]. All these species have pathogenic significance for humans, causing foodborne diseases, meningitides, wound and urinary tract infections, bacteremia, bone and joint infections, endocarditis, among others [59,60,61,62,63].

*Bacillus* spp., *E. coli* and *E. hormaechei* were already collected from fish, although they were not associated with fish diseases [64,65,66]. These three species are responsible for human diseases like food poisoning (especially, *B. cereus* and *E. coli*), bacteremia, meningitis, brain abscesses, endophthalmitis, pneumonia and sepsis [67,68,69].

There is a single report about the association of *L. adecarboxylata* with fish, more specifically in the oral cavity of sharks [70]. However, this species is frequently found in water environments. It can cause endocarditis, bacteremia and peritonitis in human hosts [71,72]. Some studies indicate that *E. hirae* could be a part of the natural microbiota of fish. There is no literature involving this bacterium in fish infections, but this species was well characterized in human infections, such as endocarditis, pyelonephritis, acute pancreatitis and septic shock, representing 1 to 3% of infections caused by *Enterococcus* spp. in clinical practice [73].

To our knowledge, this study seems to represent the first description of the species *E. acetylicum*, *K. michiganensis*, *Lelliottia* sp. and *P. vagans* associated with *S. aurata* (excluding cases where these bacteria are used as probiotics). These are environmental species, frequently found in soil, water, air, plants and insects [74,75,76,77,78,79,80]. Some of these species have been associated with infections in humans, namely immunocompromised individuals, but rarely [81,82,83,84,85,86].

Together with *E. coli*, *E. faecalis* is also an indicator of fecal contamination [87,88]. Species like *Bacillus* spp., *E. acetylicum* and *Enterococcus* spp. can be used as probiotics in aquaculture to reduce antibiotic consumption and avoid the spread of antibiotic resistance genes [66,89,90]. We could not obtain information if this was the case of this fish farming pilot station, in Portugal, possibly justifying the presence of these bacteria in our samples.

β-Lactam antibiotics are used in aquaculture in many countries, especially amoxicillin [7]. Several studies revealed a high prevalence of non-susceptibility to this class of antibiotic in fish, shrimps and water samples from aquaculture farms. These studies reported the discovery of *bla*_SHV_ and *bla*_TEM_ genes, associated with β-lactam non-susceptibility [91,92,93,94]. In our study, β-lactam resistance genes were not detected. The non-susceptibilities found here were probably related to genes or other resistance mechanisms not studied (e.g., efflux pumps). Likewise, some of the non-susceptibilities found to β-lactams are intrinsic, such as the resistance to ERT in *Pseudomonas* spp. [95]. Non-susceptibility to AMC, FOX, AZT, FEP, CTX, CAZ and PTZ in *Enterobacter* spp., *E. coli* and *Citrobacter* spp. could be explained by chromosomal AmpC β-lactamase or AmpC hyperproduction [96]. Worryingly, our study revealed decreased susceptibilities to carbapenems of *Enterobacter* spp. (ertapenem), usually associated with acquired resistance, as well as in *Pseudomonas* spp. (meropenem), which are not used in aquaculture and are considered last-resort antibiotics to treat serious infections caused by multidrug-resistant bacteria in humans [97].

Interestingly, although banned for use in food-producing animals in many countries (since 1994 in Europe; [98]), decreased chloramphenicol susceptibility continues to be described, not only in the present study (with MIC of 32 to > 64 mg/l), but also in others, with the presence of *cat* genes [87,99]. Chloramphenicol can occur naturally, produced by soil organisms such as *Streptomyces venezuelae*. The persistence of decreased susceptibility to this antibiotic may be due to intrinsic mechanisms of resistance as possibly in *P. putida* and *P. stutzeri* from our study or co-selection with other antibiotics and/or heavy metals [98,100,101]. On the other hand, florfenicol is widely used in aquaculture [8]. In a review article, Miranda et al. [102] compiled several studies in Chilean salmon farms that revealed a high frequency of decreased susceptibility to florfenicol, such as in our samples. However, there are further studies that described lower rates [103,104].

Another antibiotic class commonly used in aquaculture is quinolones, specifically flumequine and oxolinic acid [6,7]. In this study, we found low frequency of decreased susceptibility to quinolones (7% in muscles and 5% in gills, intestine and skin group). These results are confirmed by other works in *S. aurata* samples [38] and other fish species [105], while others revealed higher frequencies, also in fish samples [106]. Of the nine strains with decreased susceptibility to quinolones in this study, we found genes that justify that resistance in only two; the others may have untested resistance mechanisms. *qnrB* genes are frequently found in aquaculture environments [107,108], but to our knowledge, this is the first description of *qnrB19* gene in an *L. adecarboxylata* strain. This strain had decreased susceptibility to ciprofloxacin and was susceptible to flumequine (MIC = 4 mg/L). The *qnrB19* gene had already been reported in food-producing animals, such as pigs, poultry and veal calves, sometimes associated with mobile genetic elements, like plasmids and insertion sequences, demonstrating a potential for spreading [26,109]. Moreover, *qnrB19* gene was also found in *Enterobacteriaceae* from human clinical samples [110,111,112]. *K. pneumoniae* strain with decreased susceptibility to ciprofloxacin and susceptibility to flumequine (MIC = 1 mg/L) revealed two non-synonymous mutations in *parC* gene within the quinolone-resistance-determining regions (QRDR), one of them (G385T) already described in a *K. pneumoniae* strain from nosocomial origin [113]. It is known that mutations in QRDR of *parC* gene can result in structural changes in topoisomerase IV, reducing the affinity of this enzyme to fluoroquinolones [114].

Vancomycin and mupirocin are not used in aquaculture [7]. Decreased susceptibilities to these antibiotics were found only in muscle samples in this study. *Bacillus* spp. resistance to vancomycin probably does not represent an intrinsic resistance, since several studies indicated the susceptibility of these bacteria to this glycopeptide [67]. The search for *vanA*, *vanB* and *vanD* genes was negative, so this non-susceptibility could be explained by the presence of other genes not studied, like *vanC*, *vanE* and *vanG*, or mutations in *walKR*, *vraRS* and *graRS* genes involved in cell wall metabolism and cellular response to cell wall damage [115]. To our knowledge, this is the first description of *Bacillus* sp. strains resistant to vancomycin in *S. aurata* from aquaculture. This resistance was already found in this environment but in *Enterococcus* species [87]. Acquired reduced susceptibility to mupirocin in *S. petrasii* could be related to mutations in the *ileS* gene and, more rarely, to the acquisition of plasmid-mediated *mupA* gene, like in *S. aureus* [116]. There are no data available about the frequency of detection of decreased susceptibility to mupirocin in aquaculture.

As we demonstrated, some antibiotics used in food-producing animals are the same, or belong to the same class, as antibiotics used in humans [117]. Several antibiotics for which decreased susceptibility was found in this study are integrated into the WHO list of “critically important antimicrobials” and “highly important antimicrobials” for human medicine. This means that for some bacterial infections, these antibiotics are the only therapy available, and that they are also used in humans to treat infections caused by bacteria/resistance genes that may be transmitted from non-human sources (namely food-producing animals) [118].

## 5. Conclusions

This study unraveled some information about bacterial diversity and antibiotic resistance genes that circulate in *S. aurata* raised in aquaculture.

It is noteworthy that the difficulty of characterizing the strains as susceptible or non-susceptible due to be the absence of breakpoints to some of the species found in these aquatic environments and to antibiotics commonly used in aquaculture. Further research is needed in this area, also in relation to intrinsic resistances since opportunistic environmental bacteria can represent a health threat and a reservoir of antibiotic-resistant bacteria/resistance genes.

In the current study, we can observe protective associations in muscle samples, while positive correlations were found in gills, intestine and skin group of samples. Indeed, being part of the gills, intestine and skin group represents a risk factor for the presence of *Enterobacter* sp. and non-susceptibility to phenicols.

Antibiotics are used in humans and aquaculture to treat infections caused by bacteria. Furthermore, it was already described that antibiotic residues can remain in fish tissues for long periods of time, increasing exposure of commensal bacteria and/or fish pathogens, along with aquatic bacteria, to these antibiotics, enhancing the development of resistance [8]. Therefore, it is fundamentally a “One Health” approach, combining the efforts of human and veterinary medicine, along with agriculture sector, to reduce the spread of bacterial resistance and/or bacterial resistance genes.

## Figures and Tables

**Table 1:** Weight, furcal length, total length and condition index of the five *S. aurata* collected in March 2018, as well as the water temperature of the land tank from fish farming pilot station in the south of Portugal.

*S. aurata*	Weight (g)	Furcal Length (cm)	Total Length (cm)	Condition Index 1	Water Temperature
**Fish 1**	1303	37.7	42.3	1.72	17.5 °C
**Fish 2**	1201	34.4	38.9	2.04	
**Fish 3**	977	35.6	39.7	1.56	
**Fish 4**	1076	36.2	40.1	1.67	
**Fish 5**	1197	37.4	41.1	1.72	

^1^ Condition index allows one to assess the health of the fish through the relationship between its weight and length (values greater than 1 indicate a good condition of the fish) [9].

**Table 2:** Antibiotics used for antibiotic susceptibility testing and respective concentrations and breakpoints by bacterial family.

Family	Method	Antibiotics Tested (Concentration)	Breakpoints
***Bacillaceae***	MIC by E-test^®^	VA (0.016–256 µg/mL)	CLSI M45
***Enterobacteriaceae*** ***Erwiniaceae***	Disk diffusion	AMC (20 + 10 µg), AZT (30 µg), FEP (30 µg), CTX (5 µg), FOX (30 µg), CAZ (10 µg), ERT (10 µg), IMP (10 µg), MEM (10 µg), PTZ (36 µg), CIP (5 µg) SXT (25 µg), GEN (10 µg)	EUCAST
	MIC by broth microdilution	CHL, FLO, OTC FMQ	CLSI VET08 CASFM VET 2019
***Enterococcaceae***	Disk diffusion	AMP (2 µg), GEN HC (30 µg), STR HC (300 µg)	EUCAST
	MIC by E-test^®^	LNZ, TP, VA	
***Pseudomonadaceae***	Disk diffusion	AZT (30 µg), FEP (30 µg), CAZ (10 µg), DOR (10 µg), ERT (10 µg), IMP (10 µg), MEM (10 µg), PTZ (36 µg), CIP (5 µg), LEV (5 µg), AN (30 µg), GEN (10 µg), NET (10 µg), TMN (10 µg)	EUCAST
	MIC by broth microdilution	CHL, FLO, FMQ, OTC	CLSI M100 ^1^
***Staphylococcaceae***	Disk diffusion	FOX (30 µg), CIP (5 µg), LEV (5 µg), MOX (5 µg), RIF (5 µg), MUP (200 µg), FUS (10 µg)	EUCAST
	MIC by E-test^®^	DPC (0.016–256 µg/mL), LNZ (0.016–256 µg/mL), TP (0.016–256 µg/mL), VA (0.016–256 µg/mL)	

^1^ Breakpoints for CHL were used for FLO as well; breakpoints for CIP were used for FMQ; and breakpoints for tetracycline were used for OTC. Abbreviations—AMC: amoxicillin/clavulanic acid; AZT: aztreonam; FEP: cefepime; CTX: cefotaxime; FOX: cefoxitin; CAZ: ceftazidime; ERT: ertapenem; IPM: imipenem; MEM: meropenem; PTZ: piperacillin/tazobactam; CIP: ciprofloxacin; SXT: trimethoprim/sulfamethoxazole; GEN: gentamicin; CHL: chloramphenicol; FLO: florfenicol; OTC: oxytetracycline; FMQ: flumequine; DOR: doripenem; LEV: levofloxacin; AN: amikacin; NET: netilmicin; TMN: tobramycin; FOX: cefoxitin; MOX: moxifloxacin; RIF: rifampicin; MUP: mupirocin; FUS: fusidic acid; DPC: daptomycin; LNZ: linezolid; TP: teicoplanin; VA: vancomycin; AMP: ampicillin; GEN HC: gentamicin high concentration; STR HC: streptomycin high concentration; EUCAST: European Committee on Antimicrobial Susceptibility Testing; CLSI: Clinical and Laboratory Standards Institute; CASFM VET: Comité de l’antibiogramme de la Société Française de Microbiologie Recommandations Vétérinaires.

**Table 3:** Primers used in the detection of resistance genes that were described in this study for the first time.

Gene	Forward Primer Sequence (5’ → 3’)	Reverse Primer Sequence (5’ → 3’)	AT ^2^	PCR ^3^
***bla*_OXA-48_**	GACTATATTATTCGGGCTAA	ACCACTTCTAGGGAATAATT	58 °C	140 pb
***bla*_NDM_**	GTTTGATCGTCAGGGATGGC	AACGGTGATATTGTCACTGGT	56 °C	359 pb
***bla*_GES_**	AAAGCAGCTCAGATCGGTGT	TCTCTCCAACAACCCAATC	56 °C	707 pb
***bla*_SME_**	CAGATGAGCGGTTCCCTTTA	AACCCAATCAGCAGGAACAC	56 °C	509 pb
***qnrB*^1^**	ATGACGCCATTACTGTATAA	CTAACCAATCACCGCGATGC	49 °C	697 pb
***qnrC***	AACGTACGATCAAATTG	TCCACTTTACGAGGTTCT	55 °C	560 pb
***gyrB***	GGACAAAGAAGGCTACAGCA	CGTCGCGTTGTACTCAGATA	55 °C	880 pb
***vanA***	AAGGTCTGTTTGAATTGTCCG	CGACTTCCTGATGAATACGA	55 °C	417 bp
***vanB***	CCATACTCTCCCCGGATAGG	TTGACCTCATTTAGAACGATGC	55 °C	721 bp
***vanD***	ATTGGAATCACAAAATCCG	GGCTGTGCTTCCTGATG	55 °C	626 bp

^1^ Primers used for sequencing. ^2^ Annealing temperature. ^3^ PCR product.

**Table 4:** Bacterial families of the 136 strains recovered from muscle, gills, intestine and skin samples.

Bacterial Family	Fish Farm			
	Muscle (*n* = 5)		Gills, Intestine and Skin (*n* = 1) ^1^	
	No. of Strains	%	No. of Strains	%
***Bacillaceae***	9	10%	7	17%
***Bacillales*Family XII. *Incertae Sedis***	1	1%	0	0%
***Comamonadaceae***	2	2%	0	0%
***Enterobacteriaceae***	52	55%	25	60%
***Enterococcaceae***	4	4%	2	5%
***Erwiniaceae***	0	0%	1	2%
***Micrococcaceae***	4	4%	0	0%
***Pseudomonadaceae***	7	7%	1	2%
***Staphylococcaceae***	15	16%	6	14%
**Total (No. of strains/%)**	94	100%	42	100%

^1^ Results from gills, intestine and skin samples were treated jointly.

**Table 5:** Odds ratio (OR) and 95% confidence intervals (CI) (*p* ≤ 0.05) from the analysis of negative and positive correlations between fish samples (muscle vs. gills, intestine and skin) and each bacterial species and non-susceptibility to different antibiotics classes.

Fish Sample	Bacterial Species ^1^	Antibiotic’s Class ^2^	OR ^3^	95% CI	*p* Value
**Muscle**	ALL	Phenicols	0.3921 (P)	0.1701–0.912	≤0.01
**Gills, intestine and skin**	ALL	Phenicols	2.55	1.096–5.879	≤0.01
**Muscle**	*Enterobacter* sp.	*-*	0.1648 (P)	0.02645–0.7834	≤0.01
**Gills, intestine and skin**	*Enterobacter* sp.	*-*	6.067	1.277–37.8	≤0.01

Only significant associations are presented: *p*-values ≤ 0.05 and confidence limits excluding null values (0, 1, or [n]). ^1^ ALL include all species identified in the study, described in point 3.1. of results. ^2^ Antibiotic’s class tested was: glycopeptides, mupirocin, phenicols, phenicols, quinolones, β-lactams. ^3^ (P) indicates an OR value for a protective or negative association; otherwise, values should be interpreted as a positive association.

**Table 6:** Antibiotic susceptibility testing results of the 136 strains found in this study (these results do not include known intrinsic non-susceptibilities).

Antibiotic’s Class	Fish Farm			
	Muscle (*n* = 94)		Gills, Intestine and Skin (*n* = 42)	
	R/I (%)	S (%)	R/I (%)	S (%)
**Aminoglycosides**	0 (0)	94 (100)	0 (0)	42 (100)
**Fusidanes**	0 (0)	94 (100)	0 (0)	42 (100)
**Glycopeptides ^1^**	3 (3)	91 (97)	0 (0)	42 (100)
**Lipopeptides**	0 (0)	94 (100)	0 (0)	42 (100)
**Mupirocin**	1 (1)	93 (99)	0 (0)	42 (100)
**Oxazolidinones**	0 (0)	94 (100)	0 (0)	42 (100)
**Phenicols ^2^**	23 (24)	71 (76)	19 (45)	23 (55)
**Quinolones ^3^**	7 (7)	87 (93)	2 (5)	40 (95)
**Rifampicin**	0 (0)	94 (100)	0 (0)	42 (100)
**Tetracyclines**	0 (0)	94 (100)	0 (0)	42 (100)
**Trimethoprim/sulfamethoxazole**	0 (0)	94 (100)	0 (0)	42 (100)
**β-lactams ^4^**	12 (13)	82 (87)	4 (10)	38 (90)

^1^ Vancomycin; ^2^ chloramphenicol and florfenicol; ^3^ ciprofloxacin, flumequine and levofloxacin; ^4^ amoxicillin/clavulanic acid, aztreonam, cefepime, cefotaxime, cefoxitin, ceftazidime, ertapenem, meropenem and piperacillin/tazobactam.

**Table 7:** MIC_50_ and MIC_90_ for *Enterobacteriaceae* strains (*n* = 77).

Antibiotic	*Enterobacteriaceae*											
	Muscle (n = 52)						Gills, Intestine and Skin (n = 25)					
	MIC_50_	MIC_90_	Range	S%	I%	R%	MIC_50_	MIC_90_	Range	S%	I%	R%
Flumequine	0.5	1	0.125–2	100	NA	0	0.5	1	0.25–4	100	NA	0
Chloramphenicol	4	8	1–32	96	2	2	4	8	2–32	92	4	4
Florfenicol	8	16	1–32	56	37	7	8	16	1–32	24	60	16
Oxytetracycline	2	4	0.5–4	100	0	0	2	4	0.5–4	100	0	0

NA: not applicable, because intermediate category does not exist for flumequine.

**Table 8:** Phenotype profile of the 61 strains that revealed decreased susceptibility to at least one antibiotic, including intrinsic non-susceptibility.

Family	Species	Decreased Susceptibility Profile	No. of Strains
***Bacillaceae***	*Bacillus cereus*	VA	1
	*Bacillus* sp.	VA	2
***Enterobacteriaceae***	*Citrobacter freundii*	AMC, FOX, FLO	1
	*Citrobacter freundii* complex	AMC, FOX, FLO	1
	*Enterobacter cloacae*	AMC, FOX, CHL, FLO	2
		AMC, FOX, FLO	4
		AMC, AZT, FEP, CTX, FOX, CAZ, ERT, FLO, PTZ	1
		AMC, AZT, CAZ, ERT, FOX	1
	*Enterobacter hormaechei*	AMC, FOX, FLO	12
		AMC, FOX	5
		AMC, AZT, FEP, CTX, FOX, CAZ, ERT, FLO, PTZ	1
		AMC, AZT, FEP, CTX, FOX, CAZ, ERT, FLO, PTZ	1
	*Enterobacter* sp.	AMC, FOX, FLO	6
		AMC, FOX, FLO, ERT	1
		AMC, FOX, CAZ	1
		AMC, AZT, FEP, CTX, FOX, CAZ, CHL, ERT, FLO, PTZ	1
		AMC, AZT, CTX, FOX, CAZ, ERT, FLO	1
	*Escherichia coli*	AMC, CHL, FLO	1
		AMC, FLO	2
	*Klebsiella pneumoniae*	CIP, FLO	1
		FLO	5
	*Leclercia adecarboxylata*	CIP, FLO	1
***Pseudomonadaceae***	*Pseudomonas putida*	AZT, ERT, MEM	2
		AZT, CHL, ERT, FLO, FMQ, MEM	2
	*Pseudomonas stutzeri*	AZT, CHL, ERT, FLO, FMQ	3
		AZT, CIP, ERT, FLO, FMQ, MEM	1
***Staphylococcaceae***	*Staphylococcus petrasii*	CIP, LEV, MUP	1
**Total**			61

AMC: amoxicillin/clavulanic acid; AZT: aztreonam; FEP: cefepime; CTX: cefotaxime; FOX: cefoxitin; CAZ: ceftazidime; CHL: chloramphenicol; CIP: ciprofloxacin; ERT: ertapenem; FLO: florfenicol; FMQ: flumequine; LEV: levofloxacin; MEM: meropenem; MUP: mupirocin; PTZ: piperacillin/tazobactam; VA: vancomycin.

## Supplementary Materials

The following are available online at https://www.mdpi.com/2076-2607/8/9/1343/s1, Figure S1: Diagram summarizing the experimental design; Table S1: Odds ratio (OR) and 95% confidence intervals (CI) (*p* ≤ 0.05) from the analysis of negative and positive correlations between fish samples (muscle versus gills, intestine and skin) and each bacterial species and non-susceptibility to different antibiotic’s class (detailed results).
