# Supplementary material for: Bacterial Diversity and Antibiotic Susceptibility of Sparus aurata from Aquaculture
Source: Microorganisms. 2020 Sep 2;8(9):1343. doi: 10.3390/microorganisms8091343 (PMC7564983; doi:10.3390/microorganisms8091343)
Supplement: Supplementary file 1 [file microorganisms-08-01343-s001.zip › Figure S1.pdf]

Figure S1. Diagram summarizing the experimental design

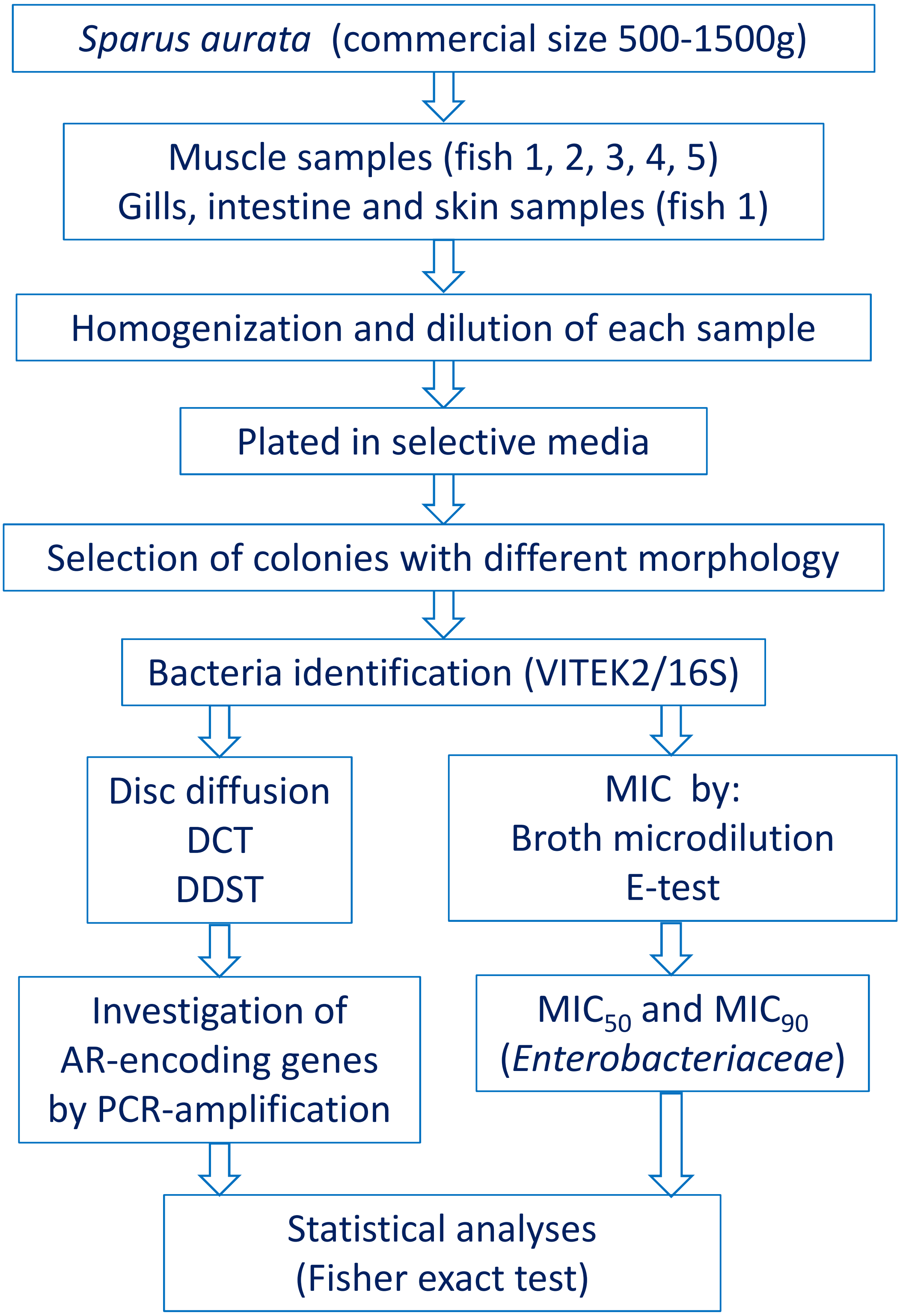

DCT, disc combination test
DDST, double disc synergy test
