# Supplementary material for: Bacterial Diversity and Antibiotic Susceptibility of Sparus aurata from Aquaculture
Source: Microorganisms. 2020 Sep 2;8(9):1343. doi: 10.3390/microorganisms8091343 (PMC7564983; doi:10.3390/microorganisms8091343)
Supplement: Supplementary file 1 [file microorganisms-08-01343-s001.zip › Figure S1.pptx]

## Slide 1
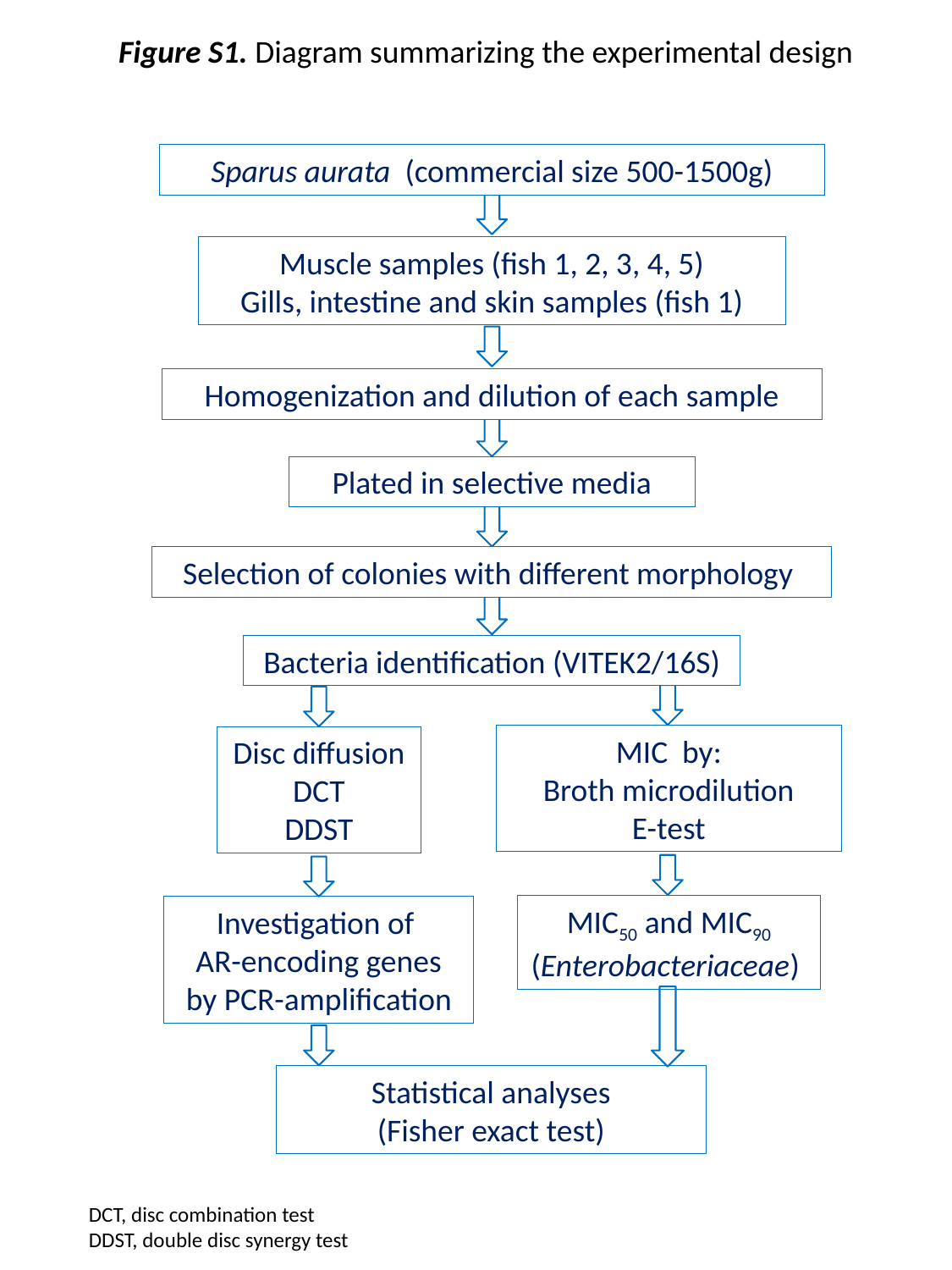

Figure S1. Diagram summarizing the experimental design
Sparus aurata (commercial size 500-1500g)
Muscle samples (fish 1, 2, 3, 4, 5)
Gills, intestine and skin samples (fish 1)
Homogenization and dilution of each sample
Plated in selective media
Selection of colonies with different morphology
Bacteria identification (VITEK2/16S)
MIC by:
Broth microdilution
E-test
Disc diffusion
DCT
DDST
MIC50 and MIC90 (Enterobacteriaceae)
Investigation of
AR-encoding genes
by PCR-amplification
Statistical analyses
(Fisher exact test)
DCT, disc combination test
DDST, double disc synergy test
