# Supplementary material for: Bacterial Diversity and Antibiotic Susceptibility of Sparus aurata from Aquaculture
Source: Microorganisms. 2020 Sep 2;8(9):1343. doi: 10.3390/microorganisms8091343 (PMC7564983; doi:10.3390/microorganisms8091343)
Supplement: Supplementary file 1 [file microorganisms-08-01343-s001.zip › Table S1.pdf]

Table S1. Odds ratio (OR) and 95% confidence intervals (CI) ($p \leq 0.05$) from the analysis of negative and positive correlations between fish samples (muscle versus gills, intestine and skin) and each bacterial species and non-susceptibility to different antibiotic's class (detailed results).

Fish sample	Bacterial family	Bacterial species	Antibiotic's class	Odds Ratio (OR)	(95% CI) lower	(95% CI) upper	p value (one-tail)	p value (two-tail)	P
Muscle	Bacillaceae	ALL	-	0.5294	0.1615	1.823	0.1831	0.3662	P
Muscle	Bacillales Family XII. Incertae Sedis	ALL	-	undefined	0.01146	undefined	0.6912	>0.9999999	
Muscle	Comamonadaceae	ALL	-	undefined	0.08359	undefined	0.4761	0.9523	
Muscle	Enterobacteriaceae	ALL	-	0.8419	0.3739	1.871	0.3949	0.7898	P
Muscle	Enterococcaceae	ALL	-	0.8889	0.1218	10.21	0.6030	>0.9999999	P
Muscle	Micrococcaceae	ALL	-	undefined	0.2952	undefined	0.2237	0.4473	
Muscle	Pseudomonadaceae	ALL	-	3.299	0.3994	152.2	0.2300	0.4600	
Muscle	Staphylococcaceae	ALL	-	1.139	0.3778	3.883	0.5123	>0.9999999	
Muscle	ALL	ALL	Glycopeptides	undefined	0.1841	undefined	0.3269	0.6538	
Muscle	ALL	ALL	Mupirocin	undefined	0.01146	undefined	0.6912	>0.9999999	
Muscle	ALL	ALL	Phenicols	0.3921	0.1701	0.912	0.01406	0.02812	
Non-Muscle	ALL	ALL	Phenicols	2.55	1.096	5.879	0.01406	0.02812	
Muscle	ALL	ALL	Quinolones	1.609	0.2879	16.5	0.4348	0.8696	
Muscle	ALL	ALL	β-lactams	1.39	0.3869	6.289	0.4108	0.8216	
Muscle	-	*Bacillus cereus*	-	0.6593	0.07282	8.204	0.4917	0.9833	P
Muscle	-	*Bacillus pumilus*	-	undefined	0.01146	'undefined	0.6912	>0.9999999	
Muscle	-	*Bacillus* sp.	-	0.5778	0.09334	4.148	0.3717	0.7434	P
Muscle	-	*Bacillus thuringiensis*	-	undefined	0.01146	'undefined	0.6912	>0.9999999	
Muscle	-	*Citrobacter freundii*	-	undefined	0.01146	'undefined	0.6912	>0.9999999	
Muscle	-	*Citrobacter freundii complex*	-	undefined	0.01146	'undefined	0.6912	>0.9999999	
Muscle	-	*Comamonas aquatica*	-	undefined	0.08359	'undefined	0.4761	0.9523	
Muscle	-	*Enterobacter cloacae*	-	3.299	0.3994	152.2	0.2300	0.4600	
Muscle	-	*Enterobacter hormaechei*	-	0.7317	0.2416	2.393	0.3596	0.7191	P
Muscle	-	*Enterobacter* sp.	-	0.1648	0.02645	0.7834	0.009823	0.01965	P
Non-Muscle	-	*Enterobacter* sp.	-	6.067	1.277	37.8	0.009823	0.01965	
Muscle	-	*Enterococcus hirae*	-	1.822	0.1725	91.9	0.5083	>0.9999999	
Muscle	-	*Exiguobacterium acetylicum*	-	undefined	0.01146	'undefined	0.6912	>0.9999999	
Muscle	-	*Klebsiella michiganensis*	-	undefined	0.08359	'undefined	0.4761	0.9523	
Muscle	-	*Klebsiella pneumoniae*	-	2.303	0.2451	111.6	0.3970	0.7939	
Muscle	-	*Kocuria rhizophila*	-	undefined	0.2952	'undefined	0.2237	0.4473	
Muscle	-	*Leclercia adecarboxylata*	-	2.407	0.7236	10.36	0.09561	0.1912	
Muscle	-	*Lelliottia* sp.	-	0.4348	0.03073	6.235	0.3634	0.7268	P
Muscle	-	*Pseudomonas putida*	-	undefined	0.2952	'undefined	0.2237	0.4473	
Muscle	-	*Pseudomonas stutzeri*	-	1.352	0.1047	72.66	0.6366	>0.9999999	
Muscle	-	*Staphylococcus aureus*	-	0.5778	0.09334	4.148	0.3717	0.7434	P
Muscle	-	*Staphylococcus capitis*	-	undefined	0.01146	'undefined	0.6912	>0.9999999	
Muscle	-	*Staphylococcus epidermidis*	-	undefined	0.01146	'undefined	0.6912	>0.9999999	
Muscle	-	*Staphylococcus haemolyticus*	-	0.6593	0.07282	8.204	0.4917	0.9833	P
Muscle	-	*Staphylococcus pasteuri*	-	0.8913	0.04525	53.85	0.6731	>0.9999999	P
Muscle	-	*Staphylococcus petrasii*	-	undefined	0.08359	'undefined	0.4761	0.9523	
Muscle	-	*Staphylococcus saprophyticus*	-	undefined	0.01146	'undefined	0.6912	>0.9999999	
Muscle	-	*Staphylococcus* sp.	-	undefined	0.01146	'undefined	0.6912	>0.9999999	
